# Novel Lateral Flow-Based Assay for Simple and Visual Detection of SARS-CoV-2 Mutations

**DOI:** 10.3389/fcimb.2022.902914

**Published:** 2022-07-14

**Authors:** Julien Gomez-Martinez, Steven Henry, Edouard Tuaillon, Philippe Van de Perre, Chantal Fournier-Wirth, Vincent Foulongne, Jean-Charles Brès

**Affiliations:** ^1^ Pathogenesis and Control of Chronic and Emerging Infections, University of Montpellier, Etablissement français du sang, INSERM, University of Antilles, Montpellier, France; ^2^ Laboratoire de Virologie, Centre Hospitalier Universitaire de Montpellier, Montpellier, France

**Keywords:** SARS-CoV-2 mutations, lateral flow strip (LFS), molecular diagnostic, visual detection, DNA microarray

## Abstract

Identification of the main SARS-CoV-2 variants in real time is of interest to control the virus and to rapidly devise appropriate public health responses. The RT-qPCR is currently considered to be the reference method to screen SARS-CoV-2 mutations, but it has some limitations. The multiplexing capability is limited when the number of markers to detect increases. Moreover, the performance of this allele-specific method may be impacted in the presence of new mutations. Herein, we present a proof-of-concept study of a simple molecular assay to detect key SARS-CoV-2 mutations. The innovative features of the assay are the multiplex asymmetric one-step RT-PCR amplification covering different regions of SARS-CoV-2 S gene and the visual detection of mutations on a lateral flow DNA microarray. Three kits (Kit 1: N501Y, E484K; Kit 2: L452R, E484K/Q; Kit 3: K417N, L452R, E484K/Q/A) were developed to match recommendations for surveillance of SARS-CoV-2 variants between January and December 2021. The clinical performance was assessed using RNA extracts from 113 SARS-CoV-2-positive samples with cycle thresholds <30, and results demonstrated that our assay allows specific and sensitive detection of mutations, with a performance comparable to that of RT-qPCR. The VAR-CoV assay detected four SARS-CoV-2 targets and achieved specific and sensitive screening of spike mutations associated with the main variants of concern, with a performance comparable to that of RT-qPCR. With well-defined virus sequences, this assay can be rapidly adapted to other emerging mutations; it is a promising tool for variant surveillance.

## Introduction

In December 2019, a cluster of pneumonia of unknown origin was reported in Wuhan, China (Wu et al., 2020; Zhu et al., 2020a). Severe acute respiratory syndrome coronavirus 2 (SARS-CoV-2), a novel coronavirus, was identified as the causative agent of coronavirus disease 19 (COVID-19). The outbreak spread rapidly across the globe and the World Health Organization, in March 2020, declared COVID-19 a pandemic (World Health Organization, 2020b). As of June 2022, more than 533 million people have been infected, resulting in over 6 million deaths (World Health Organization, 2022).

The SARS-CoV-2 genome has significantly evolved since the pandemic started, leading to the emergence of a large number of variants. Some harboring genetic mutations associated with increased transmission, virulence, and immune escape are labeled ‘variants of concern’ (VOC) and are closely monitored to assess SARS-CoV-2 variant spread. To date, five VOCs have been described, each carrying a distinct set of mutations, with key mutations mostly occurring in the receptor binding domain (RBD) of the spike (S) protein (Harvey et al., 2021).

In late 2020, B.1.1.7 (Alpha) became the first VOC (Public Health England, 2020). It carries a N501Y S mutation that confers increased transmissibility versus previous SARS-CoV-2 lineages and rapidly became the dominant strain in many countries (Davies et al., 2021; Leung et al., 2021). By early 2021, the B.1.351 (Beta) and P.1 (Gamma) variants were identified, originating from South Africa and Brazil, respectively (Tegally et al., 2020; Faria et al., 2021). Both variants contain, in addition to the N501Y mutation, the E484K mutation in the RBD of the S protein. Multiple studies reported that this mutation reduced neutralizing antibody activity, potentially resulting in immune evasion (Cele et al., 2021; Wibmer et al., 2021).

The Delta variant was first identified in India in October 2020 (Indian Ministry of Health and Family Welfare, 2021). It is part of a family of three sublineages with B.1.617.1 (Kappa) and B.1.617.3. However, only Delta is classified as a VOC by WHO. Delta is 40–60% more transmissible than the Alpha variant (Campbell et al., 2021) and was, until January 2022, the predominant SARS-CoV-2 variant. Delta harbors the L452R S mutation, associated with increased viral infectivity (Deng et al., 2021; Tchesnokova et al., 2021) and cellular immune escape (Motozono et al., 2021). The E484Q mutation is also associated with immune evasion; it is rarely present in the Delta variant but is shared by the B.1.617.1 and B.1.617.3 sublineages. Omicron (B.1.1.529) is the latest coronavirus VOC. It was first identified in Botswana and South Africa in November 2021 and is now the predominant strain worldwide. Omicron is characterized by an unusually large number of mutations, including more than 30 in the S protein and 15 in the RBD (Kumar et al., 2022).

Several studies have shown that Omicron has a significant growth advantage compared to Delta. This is mainly due to its intrinsic increased transmissibility (Pearson et al., 2021; Hui et al., 2022) and immune escape (Cao et al., 2022; Planas et al., 2022). Some of its mutations are shared with other variants. For example, N501Y (Alpha, Beta, Gamma) and K417N (Beta) contribute to higher infectivity and immune escape (Khan et al., 2021; Zhou et al., 2021). Similarly to Beta, Kappa, and B.1.617.3, Omicron harbors a substitution at residue E484 (E484A), which contributes to antibody neutralization resistance (Shah and Woo, 2022).

Global surveillance of these mutations is critical for public health decision-making. Nucleic acid amplification tests (NAATs) are the most widely used method for diagnosing acute infections. RT-qPCR of the S gene is currently considered the ‘gold standard’ method to screen for SARS-CoV-2 mutations (Carter et al., 2020). However, this technique has some limitations: it involves multiple steps and requires use of complex and expensive instruments, particularly for the detection step. Moreover, RT-qPCR is an allele-specific molecular method that might produce false-negative results in the presence of new mutations.

The COVID-19 outbreak has sparked the development of alternative NAAT strategies for fast, accurate, and affordable SARS-CoV-2 diagnostic tests (Vindeirinho et al., 2022). Among these methods, nucleic acid lateral flow immunoassays (NALFIAs) based on isothermal amplification employing a lateral flow readout have been widely developed in a point-of-care format (Zhang et al., 2022). These assays have included reverse transcription recombinase polymerase amplification (RT-RPA) (Lau et al., 2021), reverse transcription loop-mediated isothermal amplification (RT-LAMP) (Zhu et al., 2020b; Banerjee et al., 2021; Zhang et al., 2021), clustered regularly interspaced short palindromic repeats (CRISPR)-Cas-based assays (Azhar et al., 2021; Xiong et al., 2021; Zhu et al., 2021), and nucleic acid sequence based amplification (NASBA) (Wu et al., 2021). Although these tests can accurately detect SARS-COV-2, they are unable to detect several point mutations in a single assay.

**Figure 1:**
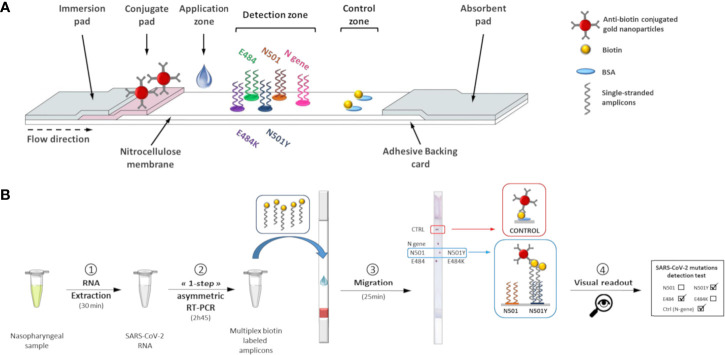
Lateral flow test for determination of SARS-CoV-2 genotypes. **(A)** Schematic view of the lateral flow strip. **(B)** Lateral flow procedure to perform visual detection of SARS-CoV-2 mutations.

Previously, our group developed a novel approach for simple and rapid multiplex detection of point mutations (single nucleotide polymorphisms, SNPs) using a combination of an asymmetric PCR amplification and a lateral flow DNA microarray strip (Gomez-Martinez et al., 2018). Gold nanoparticles, used as a reporter, allow easy visual detection of multiple nucleic acid sequences on a single device by the generation of red dots, precluding the need for expensive instruments. Here, this method was applied to the detection of key mutations within the SARS-CoV-2 S gene (**Figure 1**). Our VAR-CoV assay was designed for use in microbiology laboratories, on SARS-CoV-2 RNA-positive samples. Three mutation assay kits (Kit 1: N501Y, E484K; Kit 2: L452R, E484K, E484Q; Kit 3: K417N, L452R, E484K, E484Q, E484A) were developed to correspond with the French Ministry of Health’s recommendations for national SARS-CoV-2 testing strategies (**Table 1**). Herein, we present the analytical performance of the VAR-CoV assay and its evaluation on a panel of clinical samples from patients with suspected SARS-CoV-2 infection.

**Table 1:** French Ministry of Health’s recommendations for SARS-CoV-2 variant tracking.

Period	Feb–May 2021	May–Nov 2021	Since Dec 2021
**SARS-CoV-2 targets**	N501Y E484K	L452R E484K E484Q	L452R E484K/Q K417N or del69/70
**Main VOC targeted**	Alpha (B.1.17), Beta (B.1.351)	Alpha (B.1.17), Beta (B.1.351), Gamma (P.1)	Delta (B.1.617.2), Omicron (B.1.1.529)

VOC, variant of concern.

## Materials and Methods

### Clinical Specimens and RNA Extracts

The Centre Hospitalier Universitaire (CHU) Montpellier (Montpellier, France) (CPP Ile de France III, n°2020-A00935−34) provided 153 patient specimens tested for SARS-CoV-2. Five RNA extracts from positive samples were purchased from AlphaBio (Marseille, France). Clinical samples were collected, with informed consent, from the CHU Montpellier (Montpellier, France).

### Protocol of RT-qPCR Kits for Detection of SARS-CoV-2 Mutations

SARS-CoV-2 variant detection was performed by three RT-qPCR kits: ID™ SARS-CoV-2/N501Y/E484K Quadruplex, ID™ SARS-CoV-2/VOC evolution Pentaplex, and ID™ SARS-CoV-2/VOC Revolution Pentaplex (ID Solutions, Grabels, France) according to the manufacturer’s instructions. The last assay targets three viral genes (N, RdRP, and S) and screens for the presence of the K417N, L452R, E484K, and E484Q mutations.

Viral nucleic acid was extracted from 200 µL of sample preservation solution using the EZ1 Virus Mini Kit v2.0 (Qiagen, Hilden, Germany) and EZ1^®^ Advanced XL instrument and eluted in 60 µL elution buffer. RNAs were stored at -80°C until their use. The RT-qPCR tests were performed on a LightCycler^®^ 480 system (Roche Diagnostics, Rotkreuz, Switzerland). To avoid biasing the variant screening, a cut-off cycle threshold (Ct) value was fixed; all tests with Ct values above 30 were excluded (Roquebert et al., 2021).

### Multiplex Asymmetric One-Step RT-PCR

Based on Wuhan-Hu-1 SARS-CoV-2 reference sequence (*NC_045512.2*), and on conserved and variable genomic regions of the virus (Abeer et al., 2021), we designed specific primers (**Table S1**) using OligoAnalyzer 3.1 (Integrated DNA Technologies, Coralville, USA).

For Kit 1, a first multiplex asymmetric RT-PCR was designed to co-amplify one region in the N gene and one in the S glycoprotein gene. A single S-specific primer set was designed to flank the N501Y and E484K mutations, resulting in co-amplification of the two mutations in the same fragment. To match the French Ministry of Health’s recommendations for national SARS-CoV-2 testing strategies in May 2021 (Ministère des Solidarités et de la Santé, 2021b) and in December 2021 (Ministère des Solidarités et de la Santé, 2021a), a second multiplex PCR protocol based on the PCR for Kit 1 was developed for Kit 2 and Kit 3 by adding a new primer set targeting the L452R and K417N mutation sites in the same amplicon.

Each RT-PCR reaction contained 1X QIAGEN OneStep RT-PCR buffer (Qiagen, Germany), 400 µM dNTPs, 1 µM biotin-labeled excess primers, 0.1 µM limiting primers, 2 µl QIAGEN OneStep RT-PCR enzyme mix, and 5 µL RNA extract. Multiplex asymmetric PCR was performed in a Biometra TProfessional thermal cycler (Analytik Jena, Jena, Germany) starting with a 30-min reverse transcription step at 50°C and a 15-min Taq polymerase activation at 95°C, followed by 55 cycles of 40 s at 94°C, 40 s at 55°C, and 30 s at 72°C, and a final extension step of 10 min at 72°C.

### Preparation of Lateral Flow Test Strips

The dry reagent dipstick containing a wicking pad, a glass-fiber conjugate pad, a nitrocellulose membrane, an absorbent pad, and an adhesive backing card was assembled as previously described (Gomez-Martinez et al., 2018). All components were purchased from Merck Millipore (Darmstadt, Germany). The lateral flow test strip was composed of a test zone and a control zone. The probes were manually spotted on the membrane in spotting buffer (6X saline-sodium citrate (SSC), 2% methanol, 2% sucrose) with a final volume of 0.1 µL at selected concentrations (**Table S2**). These probes were designed using OligoAnalyzer 3.1 (IDT DNA) with the allele-specific SNPs placed in central position to enhance allele discrimination. Three types of test strips were designed for the detection of three sets of SARS-CoV-2 mutations (**Figure S1**).

Probes corresponding to the wild-type (WT) sequences were spotted on the left side of the membrane, while probes corresponding to the mutant (MUT) sequences were spotted on the right side. For Kit 2, which permits detection of E484K/Q mutations, a new level of detection was added to the test strip by spotting the E484Q specific probe above the E484/E484K probes. For Kit 3, four levels of detection were necessary to detect K417N, L452R, and E484K/Q/A mutations where the last level included the E484Q and E484A probes.

A nucleocapsid (N)-gene probe was spotted on the upper part of the test zone to act as a PCR positive control. A solution of 25 µg/mL biotinylated BSA in spotting buffer (0.1 µL) was spotted in duplicate at the top of the strip to define a control zone. After assembly, the test strips were stored dry in a desiccator cabinet.

### Visual Detection of SARS-CoV-2 S-Gene Mutations by Lateral Flow Dipstick

Without purification or denaturation, biotin-labeled amplicons (1.5 µL) were applied to the bottom of the membrane, above the conjugate pad. The wicking pad was then immersed in a 50 mL conical tube containing prewarmed running solution (1X SSC, 10% Tween-20, 0.5% sodium dodecyl sulfate, and 1 M urea) and incubated at 52°C in a hybridization incubator (Tech, Cole-Parmer, Staffordshire, UK). Visual detection of red dots on the control zone allows validation of the assay. The result of the sample can be determined from the positions of the red dots on the detection zone (**Figure 1B**) by using the interpretation guide provided.

### Analytical Performance of the VAR-CoV Assay

To evaluate the capacity of the VAR-CoV Kit 1 assay to detect and discriminate between alleles for each mutation (N501Y, E484K), nine SARS-CoV-2-positive samples were tested: three WT (N501, E484), three harboring N501Y mutations, and three harboring both mutations (N501Y, E484K). Three SARS-CoV-2 negative samples were used as negative controls.

Analytical sensitivities of Kit 1 and Kit 3 were determined using the RNA controls with a well-quantified viral load. The samples were serially diluted in molecular grade, nuclease-free water (2,800; 560; 112; 56; 28; 14; 7; 3.5; 1.75; and 0.9 copies/reaction). For Kit 1, AMPLIRUN^®^ SARS-CoV-2 RNA control and AMPLIRUN^®^ SARS-CoV-2 Beta (B.1.351) RNA control (Vircell, Granada, Spain) were tested, whereas Kit 3 was evaluated on three MUT templates: one harboring E484A and K417N mutations (AMPLIRUN^®^ SARS-CoV-2 Omicron RNA control), one harboring the L452R mutation (AMPLIRUN^®^ SARS-CoV-2 Delta RNA control), and one harboring K417N and E484K mutations (AMPLIRUN^®^ SARS-CoV-2 Beta RNA control). In parallel, the analytical specificity of our variant screening assay was evaluated on a panel of respiratory viruses: HCoV-229E, HcoV-OC43, SARS-CoV-1, MERS-CoV, Influenza A-H1N1, Influenza A-H3N1, and Influenza B.

### Evaluation of the VAR-CoV Assay on Clinical Samples

The clinical performance of the three VAR-CoV assay kits was assessed using RNA extracts from 113 positive samples with Cts<30. In accordance with the protocol described in the previous sections, 45 samples were processed with Kit 1, 39 samples with Kit 2, and 29 samples with Kit 3. Discordant results between the reference method and the VAR-CoV assay were compared with whole genome sequences.

## Results

### VAR-CoV Assay Detection of SARS-CoV-2

The VAR-CoV assay we developed works in the following way: co-amplification of N- and S-gene specific fragments was performed by multiplex asymmetric one-step RT-PCR to produce 5’-biotinylated single-stranded products. Amplicons were applied to the ready-to-use lateral flow dipstick and migration was achieved in a dry oven. Under the effect of capillary migration, the dried anti-biotin conjugated gold nanoparticles were rehydrated with the running buffer and released from the conjugate pad. As the solution moved along the membrane, the amplicons migrated and hybridized to their complementary allele-specific probes in the detection zone. Hybridized biotinylated amplicons were detected by anti-biotin antibodies conjugated to gold nanoparticles. Aggregation of gold nanoparticles enabled visual detection as red spots. The excess of gold nanoparticles was captured at the control zone of the dipstick by immobilized biotinylated BSA, forming two red dots that indicated the proper reagent flow in each device. After an assay was completed, the interpretation guide could be used to determine the SARS-CoV-2 genotype of the sample based on the visual detection of dots. The validity of an assay was determined by the two following criteria: the generation of two red dots on the control zone and at least one positive signal for each mutation.

### Visual Detection of SARS-CoV-2 Mutations

**Figure 2:**
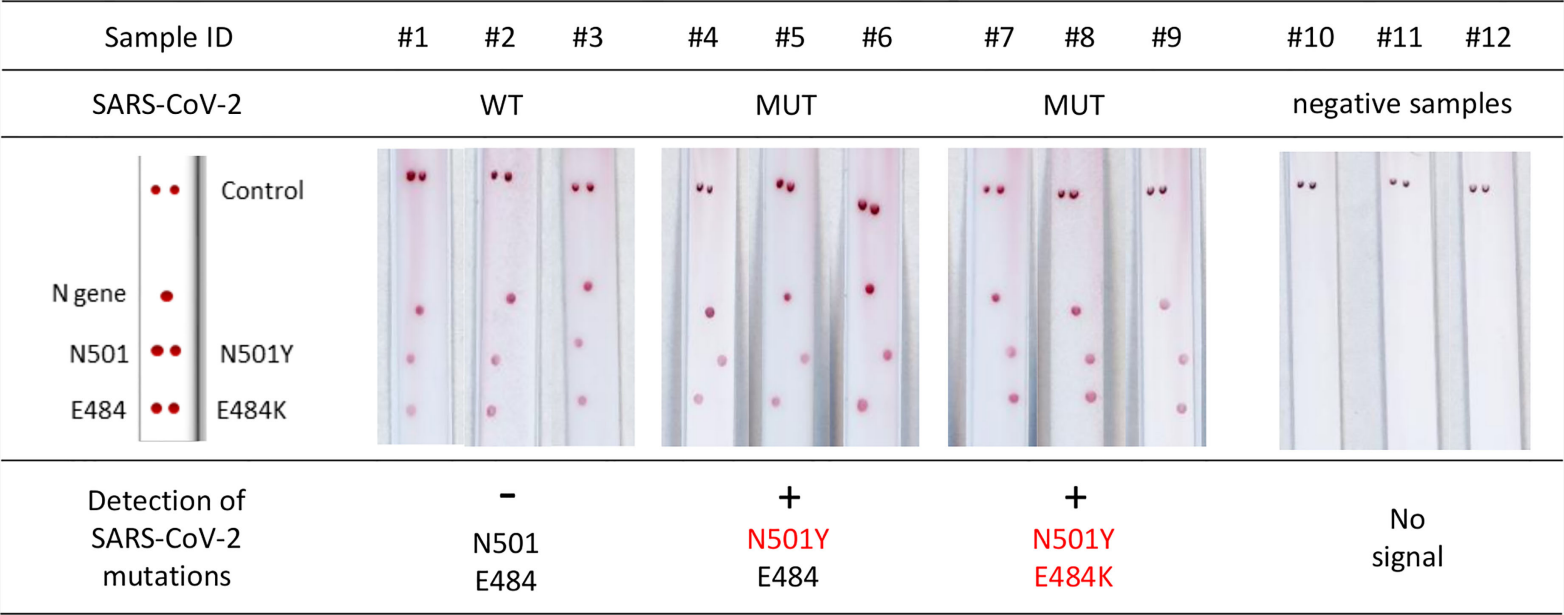
Evaluation of the VAR-CoV assay for detecting key mutations from Kit 1 (N501Y, E484K). Evaluation was performed on nine samples including: three wild-type SARS-CoV-2 samples (#1 to #3), three N501Y samples (#4 to #6), three E484K/N501Y samples (#7 to #9), and three negative SARS-CoV-2 samples (#10 to #12). Visual detection of biotinylated amplicons and genotyping were performed using the interpretation guide (on the left).

Use of Kit 1 on nine samples from patients with confirmed SARS-CoV-2 infection resulted in intense and specific signals (**Figure 2**). There was a 100% concordance rate between the VAR-CoV assay and the RT-qPCR reference method for correctly detecting N501Y and E484K mutations. No nonspecific signals were observed.

### Limit of Detection

**Figure 3:**
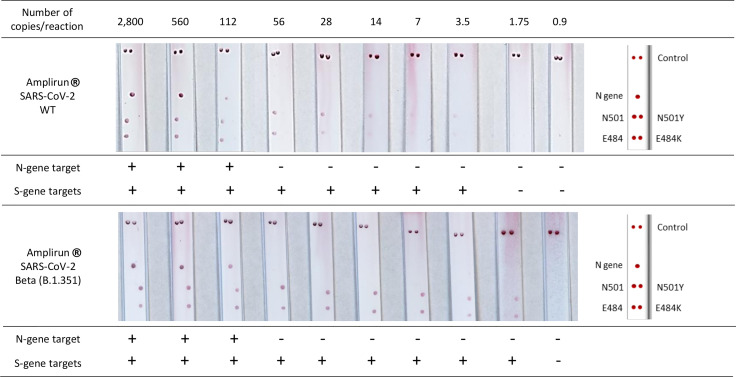
Determination of the limit of detection of the VAR-CoV assay Kit 1 on a serial dilution of the AMPLIRUN^®^ SARS-CoV-2 wild-type and AMPLIRUN^®^ SARS-CoV-2 Beta (B.1.351) RNA controls. Visual detection of biotinylated amplicons was performed using the interpretation guide (on the right). WT, wild-type.

The serial dilution of the RNA controls gave strong signals on the N-gene and S-gene probes without nonspecific signals on other capture probes. The VAR-CoV assay Kit 1 detected the N-gene and S-gene targets with a limit of detection (LOD) of 112 copies/reaction and 3.5 copies/reaction for WT templates. For E484K and N501Y S-gene mutations, the LOD was 1.75 copies/reaction (**Figure 3**). The LOD of Kit 3 for detecting N-gene targets was 560 copies/reaction, for detecting E484K and L452R mutations was 7 copies/reaction, and for detecting E484A mutations was 14 copies/reaction. The LODs for detecting K417N mutations were 7 and 14 copies/reaction for the AMPLIRUN SARS-Cov2 Omicron and Beta RNA controls, respectively (**Figure S2**).

### Diagnostic Accuracy

**Figure 4:**
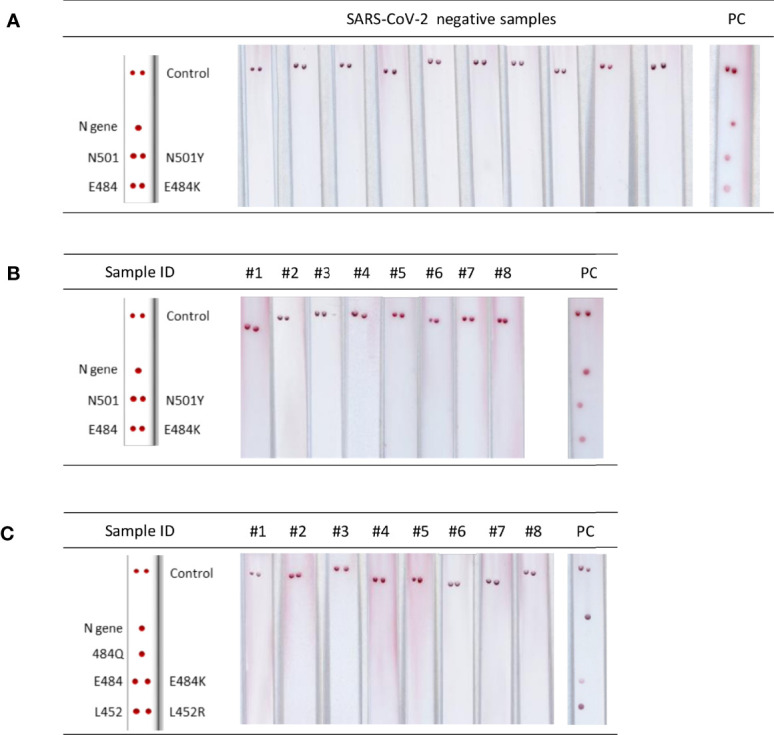
Analytical performance of the VAR-CoV assay. **(A)** Images of lateral flow strips after migration of PCR products from negative samples. PC, positive control **(B, C)** Evaluation of cross-reactivity for the two primer sets [PCR Kit 1**(B)** and PCR Kit 2/3**(C)**] against respiratory viruses. Kits 2 and 3 use the same set of primers. #1: human coronavirus (HCoV)-OC43; #2: HCoV-229E; #3: SARS-CoV-1; #4: Middle East respiratory syndrome coronavirus (MERS-CoV); #5: influenza A H1N1; #6: influenza A H3; #7: influenza B; #8: no template control; PC, positive control. Visual detection of biotinylated amplicons was performed using the interpretation guide (on the left).

The VAR-CoV assay showed 100% diagnostic specificity when tested with SARS-CoV-2-negative samples (**Figure 4A**) and 100% analytical specificity on samples infected with other respiratory viruses (**Figures 4B, C**). No cross-reactivity towards the seven respiratory viruses was observed; for the negative samples, no nonspecific signals appeared on any probes.

### Clinical Evaluation of the VAR-CoV Assay on Patient Samples

To validate the three SARS-CoV-2 mutation assay kits, we analyzed RNA extracts from 113 SARS-CoV-2-positive samples and detected 112 out of 113 positive samples (99.1%), with no false-positive signals on negative samples. Among the positive samples, we detected 21 N501Y mutations and nine E484K mutations with Kit 1 (**Table 2**), 34 L452R mutations, four E484K mutations, and five E484Q mutations with Kit 2 (**Table 3**), and 29 K417N mutations and 29 E484A mutations with Kit 3 (**Table 4**). One sample remained invalid; no signal in the detection zone and intense signals in the control zone indicated a PCR amplification failure. The RT-qPCR screening kit correctly analyzed the sample, which harbored an N501Y mutation. We also observed two discrepancies between the VAR-CoV assay and the reference method. For one sample, RT-qPCR returned an undetermined result, whereas our assay detected an N501Y mutation. For another sample, L452R and E484Q mutations were not detected with the ID Solutions kit but were correctly detected by our assay. Both discrepancies were analyzed by comparison with the available whole genome sequences, which confirmed the VAR-CoV assay conclusions. For the samples identified as being infected with the Omicron variant, the ID Solutions kit was not able to give information on the 484 mutation site, but our assay detected the E484A mutation on all 29 samples. Results were confirmed by Sanger sequencing of the RBD.

**Table 2:** Results of the VAR-CoV Kit 1 assay (N501Y, E484K) on clinical samples.

Target in spike (S) gene	Number of tested samples	Allele/mutation	ID Solutions kit (RT-qPCR)^a^		VAR-CoV assay			No. of discordances
			No. (%) of genotyped samples	No. (%) of undetermined samples	No. (%) of genotyped samples		No. (%) of invalid samples	
501	45	N501 N501Y	nt 21 (95.5)	- 1 (4.5)	23 (100) 21 (95.5)		0 (0) 1 (4.5)	- 2 ^b^
484	45	E484 E484K	nt 9 (100)	- 0 (0)	36 (100) 9 (100)		0 (0) 0 (0)	- 0

nt, not tested. ^a^Results determined with ID™ SARS-CoV-2/N501Y/E484K Quadruplex. ^b^Two discordances were observed: one sample remained invalid with the VAR-CoV assay whereas it was correctly analyzed with the RT-qPCR screening kit. A second sample was correctly analyzed with the VAR-CoV assay whereas the reference method returned an undetermined result. This discrepancy was analyzed by comparison with the available whole genome sequences, which confirmed the VAR-CoV assay conclusions.

**Table 3:** Results of the VAR-CoV Kit 2 assay (L452R, E484K/Q) on clinical samples.

Target in spike (S) gene	Number of tested samples	Allele/mutation	ID Solutions kit (RT-qPCR)^a^		VAR-CoV assay		No. of discordances
			No. (%) of genotyped samples	No. (%) of undetermined samples	No. (%) of genotyped samples	No. (%) of invalid samples	
484	39	E484 E484K E484Q	nt 4 (100) 4 (75)	- 0 (0) 1 (0)	30 (100) 4 (100) 5 (100)	0 (0) 0 (0) 0 (0)	- 0 1^b^
452	39	L452 L452R	nt 33 (97.1)	- 1 (0)	5 (100) 34 (100)	0 (0) 0 (0)	- 1^b^

nt, not tested. ^a^Results determined with ID™ SARS-CoV-2/VOC Evolution Pentaplex. ^b^Discrepancy was analyzed by comparison with the available whole genome sequences, which confirmed the VAR-CoV assay conclusions.

**Table 4:** Results of the VAR-CoV Kit 3 assay (K417N, L452R, E484K/Q/A) on clinical samples.

Target in spike (S) gene	Number of tested samples	Allele/mutation	ID Solutions kit (RT-qPCR)^a^		VAR-CoV assay		No. of discordances
			No. (%) of genotyped samples	No. (%) of undetermined samples	No. (%) of genotyped samples	No. (%) of invalid samples	
484	29	E484 E484K/Q E484A	nt 0 (0) nt	- 0 (0) 0 (0)	0 (0) 0 (0) 29 (100)^b^	0 (0) 0 (0) 0 (0)	- 0 0
452	29	L452 L452R	nt 0 (0)	- 0 (0)	29 (100) 0 (0)	0 (0) 0 (0)	- 0
417	29	K417 K417N	nt 29 (100%)	- 0 (0)	0 (0) 29 (100)	0 (0) 0 (0)	- 0

nt, not tested. ^a^Results determined with ID™ SARS-CoV-2/VOC Revolution Pentaplex. ^b^Results confirmed by Sanger sequencing of the receptor binding domain.

**Figure 5:**
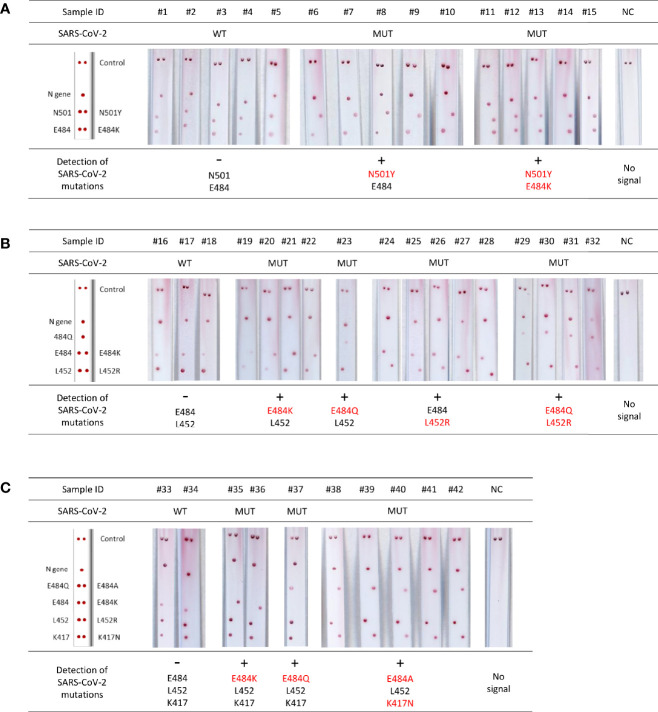
Validation of VAR-CoV assay for screening for mutations on clinical specimens. **(A)** Validation of VAR-CoV mutation assay Kit 1 on wild-type SARS-CoV-2 positive samples (#1 to #5) and samples harboring N501Y (#6 to #10) and E484K/N501Y spike mutations (#11 to #15). **(B)** Validation of VAR-CoV mutation assay Kit 2 on wild-type samples (#16 to #17) and samples harboring E484K (#19 to #22), E484Q (#23), L452R (#24 to #28), and L452R/E484Q mutations (#29 to #32). **(C)** Validation of VAR-CoV mutation assay Kit 3 on wild-type samples (#33, #34) and samples harboring E484K (#35, #36), E484Q (#37), and K417N/E484A mutations (#38 to #42). NC, negative control. Visual detection of biotinylated amplicons was performed using the interpretation guide (on the left).

The performance of the test was assessed using positive clinical samples; results showed a 99.1% sensitivity and 100% specificity when compared with the reference RT-qPCR assay for the detection of SARS-CoV-2 mutations (**Figure 5**).

## Discussion

Characterizing the main SARS-CoV-2 variant spread in real time is of interest to control the virus and rapidly devise appropriate public health responses. Here, we report the development of a simple molecular method that identifies SARS-CoV-2 key mutations using a ready-to-use lateral flow DNA strip. Our first mutation assay kit (Kit 1) detected two mutations (N501Y, E484K) present in the first established VOCs: the B.1.1.7 (Alpha), B.1.351 (Beta), and P.1 (Gamma) lineages. With the emergence of new mutations present in different VOCs and new variants of interest, the French recommendations for mutation screening evolved in May 2021. In the context of the predominant B.1.1.7 variant (>80% of infections), screening for N501Y was removed. The new strategies focused on key mutations with a clinical impact, such as increased transmissibility (L452R) (Motozono et al., 2021) or immune evasion (K417N, E484K/Q) (Cherian et al., 2021; Garcia-Beltran et al., 2021). In two weeks, we rapidly adapted our assay to this new panel of mutations (Kit 2) by designing relevant primers and probes. In December 2021, with the appearance of the highly transmissible B.1.1.529 variant, our Kit 2 assay was updated (Kit 3) by adding new mutations (K417N, E484A) that can impair neutralizing antibodies. After the development of new capture probes, we were able to detect the K417N, L452R, and E484K/Q/A mutations while keeping the PCR amplification protocol used for Kit 2. Thanks to the high multiplexing capacity of the VAR-CoV assay to detect multiple mutations in one amplicon, with three PCR fragments we were able to detect four targets and nine alleles in a single test.

Results obtained with both kits showed that the VAR-CoV assay is a sensitive assay with a high level of concordance with standard RT-qPCR assays for detecting N-gene and S-gene mutations. A slight decrease in sensitivity was observed for results obtained with Kit 3 versus those obtained with Kit 1. This can be explained by the increase of mutations analyzed in the assay and the number of PCR primers required in the multiplex PCR mix. These levels of sensitivity are similar to those reported for RT-qPCR assays (Fung et al., 2020; Giri et al., 2021). Moreover, the assay showed no cross-reactivity with other respiratory pathogens.

For proof of concept, our assay was used to detect mutations in clinical samples with a PCR Ct <30 to ensure a high level of sensitivity – samples with high Ct are often at or near the limit of detection and can complicate result interpretation (Rhoads and Pinsky, 2021). When results were compared with sequence data, VAR-CoV assay sensitivity was close to 100% (99.1%) in 113 samples previously confirmed to be SARS-CoV-2. The only assay failure may be the result of RNA degradation during storage or multiple freeze–thaw cycles (Holohan et al., 2021). The specificity was 100% based on the analysis of 40 negative clinical specimens or samples positive for other respiratory viruses. Furthermore, the VAR-CoV assay has many strengths. It provides a simple visual readout and can be easily implemented in laboratories already performing NAATs. It also avoids the risk of false-negative results in the event of lower sensitivity, by using samples with a PCR Ct <30, or in the event of alternate mutations (e.g., E484Q) within the probe-binding site. Our assay screens for the presence of WT and mutated sequences in a single test, whereas allele-specific RT-qPCR can have unequal sensitivities within its various mutated targets, which may result in an underestimation of the proportion of variant strains and false-negative results in the presence of new mutations.

According to WHO recommendations for molecular testing of SARS-CoV-2 infection, the VAR-CoV-assay has the potential to be used as a first-line test and to screen for key mutations (Mardian et al., 2021). A minimum of two independent targets are required (World Health Organization, 2020a); our mutation assays, Kit 2 and 3, target three and four regions of the SARS-CoV-2 virus, respectively.

The time-to-result was around 3 h 40, but further improvements are already in progress to reduce this to 2 h. The test was designed to be user-friendly and for its use as a unitary assay. It is more suited for low-throughput analysis in delocalized laboratories. Our assay is affordable: the cost for mutations screening, including viral RNA extraction and all reagents, is below $12. Therefore, it has the potential to be implemented in laboratories in low-resource settings, to provide access to a simple and affordable SARS-CoV-2 mutation screening assay (Norton et al., 2021).

The assay has some limitations. First, the process workflow requires opening tubes containing PCR amplicons for their transfer to the strip. To prevent cross-contamination, it is necessary to centrifuge tubes before opening and the post-PCR step must be performed in a PCR workstation. In addition, human errors might occur during visual inspection of the lateral flow tests and result interpretation. Developing software able to automatically analyze the strips could improve result accuracy and traceability.

In conclusion, we have developed a simple molecular method to screen for the main SARS-CoV-2 mutations, which displayed high sensitivity and specificity on clinical samples. This assay could be easily and rapidly adapted for new mutations of SARS-CoV-2, but also developed for new emerging or already established infectious diseases.

## Data Availability Statement

The original contributions presented in the study are included in the article/**Supplementary Material**. Further inquiries can be directed to the corresponding author.

## Author Contributions

JG-M and SH conducted experiments and collected data (patient information); JG-M, SH, ET, PP, CF-W, VF, and J-CB developed methodology; JG-M, SH, ET, VF, and J-CB analyzed and interpreted data; SH, ET, PP, CF-W reviewed the manuscript; J-GM, VF, and J-CB wrote and reviewed the manuscript; J-CB edited the manuscript; VF and J-CB designed and supervised the study. All authors have read and approved the final version of the manuscript.

## Funding

This study was financially supported by the Direction de la Recherche et de la Valorisation of the Etablissement Français du Sang.

## Conflict of Interest

The authors declare that the research was conducted in the absence of any commercial or financial relationships that could be construed as a potential conflict of interest.

## Publisher’s Note

All claims expressed in this article are solely those of the authors and do not necessarily represent those of their affiliated organizations, or those of the publisher, the editors and the reviewers. Any product that may be evaluated in this article, or claim that may be made by its manufacturer, is not guaranteed or endorsed by the publisher.
