# Supplementary figures and images for: Novel Lateral Flow-Based Assay for Simple and Visual Detection of SARS-CoV-2 Mutations
Source: Front Cell Infect Microbiol. 2022 Jul 14;12:902914. doi: 10.3389/fcimb.2022.902914 (PMC9329616; doi:10.3389/fcimb.2022.902914)

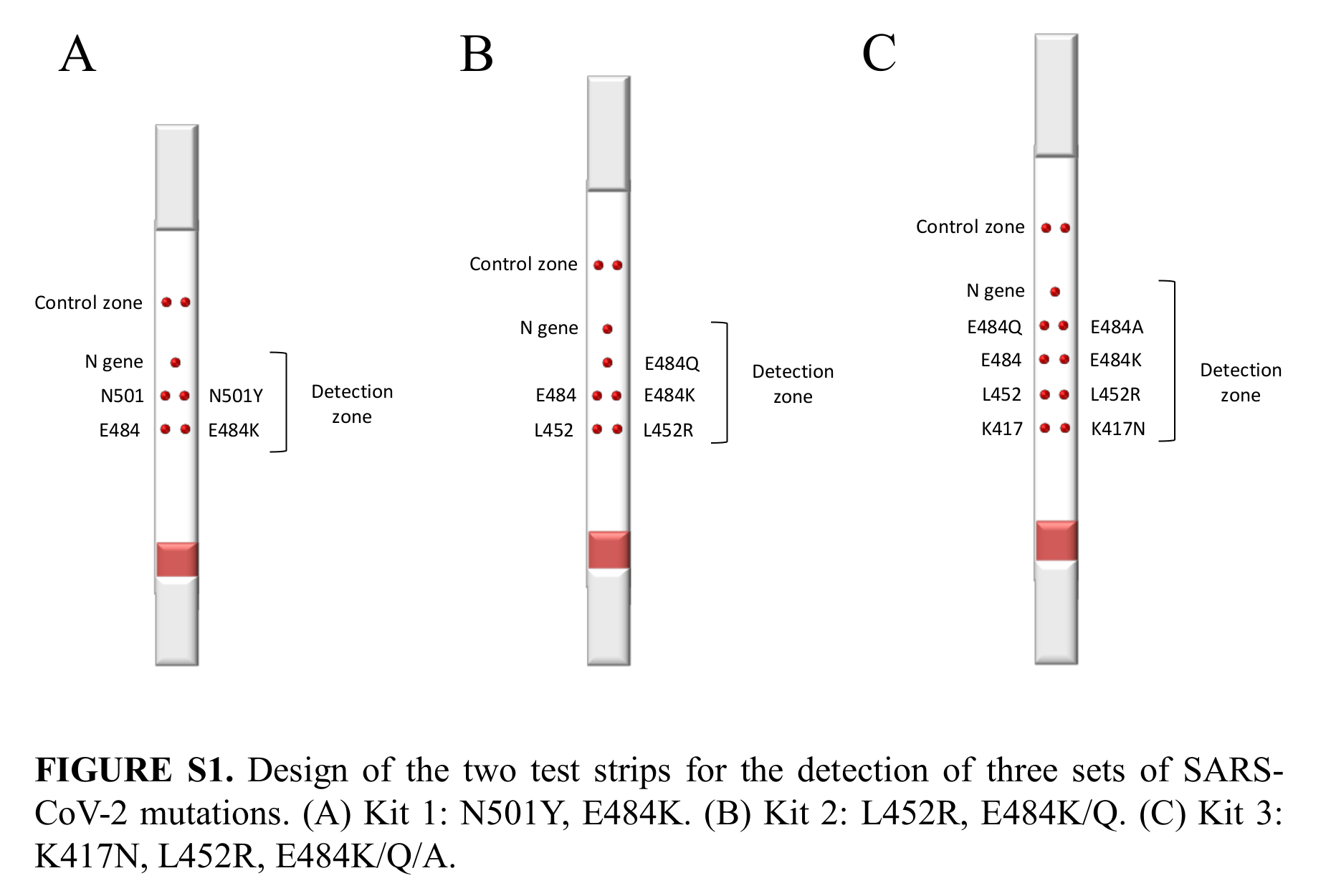

Supplement: Supplementary file 1 [file Image_1.tif]

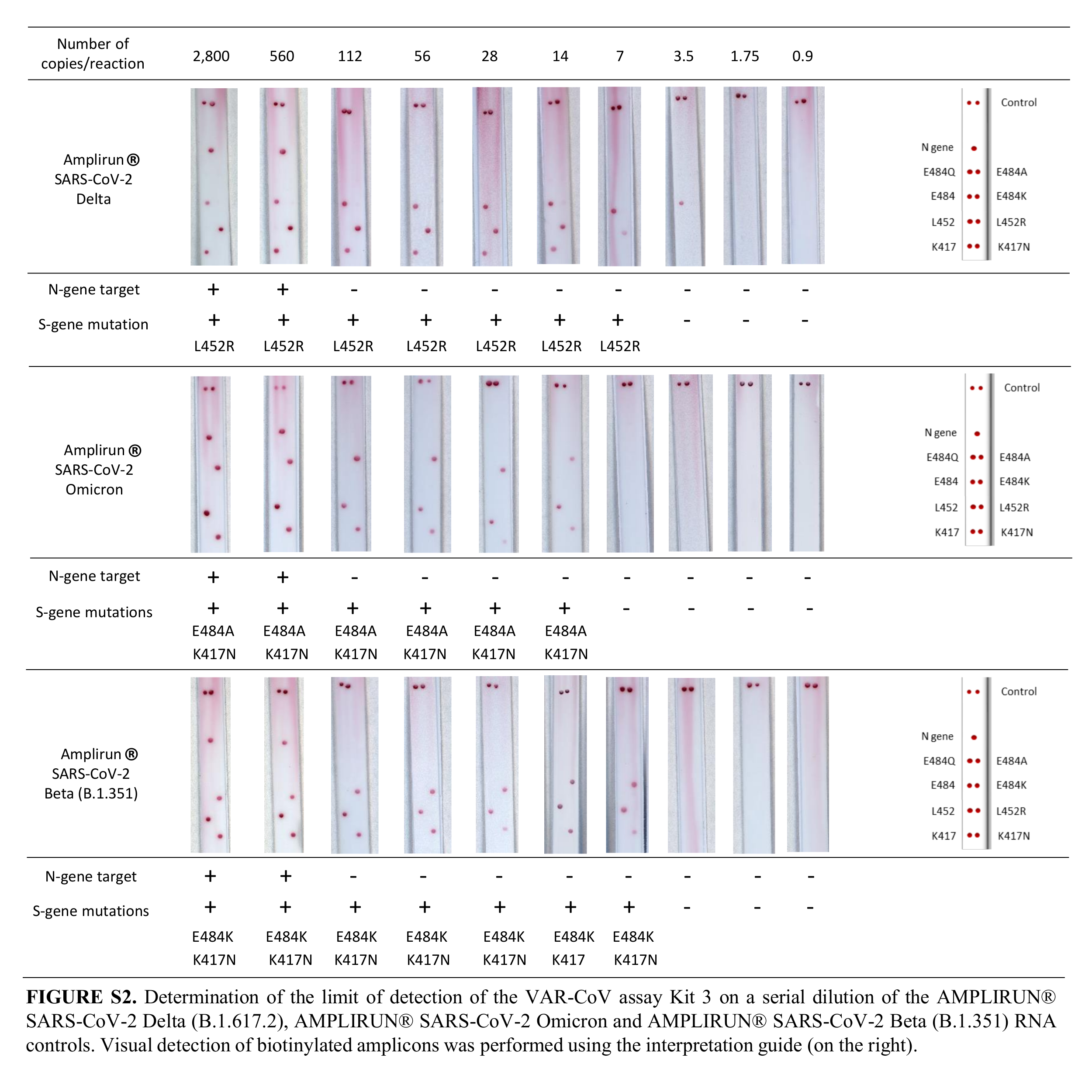

Supplement: Supplementary file 2 [file Image_2.tif]
