# Supplementary material for: Novel Lateral Flow-Based Assay for Simple and Visual Detection of SARS-CoV-2 Mutations
Source: Front Cell Infect Microbiol. 2022 Jul 14;12:902914. doi: 10.3389/fcimb.2022.902914 (PMC9329616; doi:10.3389/fcimb.2022.902914)
Supplement: Supplementary file 3 [file Table_1.docx]

**SUPPLEMENTAL TABLES**

**TABLE S1.** Primers used for the multiplex asymmetric one step RT-PCR amplification.

| **SARS-CoV-2 target** | | **Primer sequences** | **Conc.**  **(µM)** | ***T_m_***  **(°C)** | **PCR**  **Kit 1** | **PCR**  **Kit 2 &**  **Kit 3** |
| --- | --- | --- | --- | --- | --- | --- |
| **N gene** | N-control | 5′- Biotin-GTAAACCTTGGGGCCGA-3′ | 1 | 57.2 |  |  |
|  |  | 5′-CCGCATTACGTTTGGTGGACCCTCAG-3′ | 0.1 | 62.3 |  |  |
| **S gene** | E484K/Q/A, N501Y | 5′-Biotin-CAACTGAAATCTATCAGGCCGGTA-3′ | 1 | 58.2 |  |  |
|  |  | 5′-AGGTCCACAAACAGTTGCTGGTGCA-3′ | 0.1 | 62.1 |  |  |
|  | K417N, L452R | 5′-GAGGTGATGAAGTCAGACAAATCGCTCCAGG-3′ | 0.1 | 62.3 |  |  |
|  |  | 5′-Biotin-CAAAAGGTTTGAGATTAGACTTCCTAAACAATC-3′ | 1 | 57.6 |  |  |

**TABLE S2.** Capture probes spotted onto the membrane. The bold and underlined nucleotides indicate the SNPs in the allele-specific probes.

| **SARS-CoV-2 target** | **Allele** | **Probe sequences** | **Conc. (µM)** | ***T_m_* (°C)** |
| --- | --- | --- | --- | --- |
| **N gene** | N-control | 5′- C_6_ NH_2_-(dT)_15_CAACTGGCAGTAACCAG-3′ | 50 | 50.3 |
| **S gene** | K417 | 5′- C_6_ NH_2_-(dT)_15_AAACTGGAAA**G**ATTGCTGA-3' | 100 | 49.4 |
|  | K417N | 5′- C_6_ NH_2_-(dT)_15_AAACTGGAAA**T**ATTGCTGATT -3′ | 100 | 48.1 |
|  | L452 | 5′- C_6_ NH_2_-(dT)_15_AATTATAATTACC**T**GTATAGATTGTTTA-3′ | 50 | 47.3 |
|  | L452R | 5′- C_6_ NH_2_-(dT)_15_TTATAATTACC**G**GTATAGATTGTT-3′ | 50 | 47.4 |
|  | E484 | 5′- C_6_NH_2_-(dT)_15_TTAAAACCTT**C**AACACCATTA-3′ | 200 | 47.6 |
|  | E484K | 5′- C_6_ NH_2_-(dT)_15_ATTAAAACCTT**T**AACACCATTAC -3′ | 100 | 47.4 |
|  | E484Q | 5′- C_6_ NH_2_-(dT)_15_CATTAAAACCTT**G**AACACCATTACA -3′ | 200 | 52.2 |
|  | E484A | 5′- C_6_ NH_2_-(dT)_15_ATTAAAACCT**G**CAACACCATT-3′ | 100 | 51 |
|  | N501 | 5′- C_6_ NH_2_-(dT)_15_CAACACCAT**T**AGTGGGTTG-3′ | 100 | 51.2 |
|  | N501Y | 5′- C_6_NH_2_-(dT)_15_CAACACCAT**A**AGTGGGTTG -3′ | 100 | 51.2 |

SNP, single nucleotide polymorphism
